# Apoptotic Response through a High Mobility Box 1 Protein-Dependent Mechanism in LPS/GalN-Induced Mouse Liver Failure and Glycyrrhizin-Mediated Inhibition

**DOI:** 10.1371/journal.pone.0092884

**Published:** 2014-04-01

**Authors:** Noriyuki Kuroda, Kouji Inoue, Tadayuki Ikeda, Yaiko Hara, Kenjiro Wake, Tetsuji Sato

**Affiliations:** 1 Department of Anatomy and Histocytology, School of Dental Medicine, Tsurumi University, Yokohama, Japan; 2 Research Center of Electron Microscopy, School of Dental Medicine, Tsurumi University, Yokohama, Japan; 3 Department of Geriatric Dentistry, School of Dental Medicine, Tsurumi University, Yokohama, Japan; 4 Liver Research Unit, Minophagen Pharmaceutical Co. Ltd., Tokyo, Japan; University of Navarra School of Medicine and Center for Applied Medical Research (CIMA), Spain

## Abstract

HMGB1 is a nuclear component involved in nucleosome stabilization and transcription regulation, but extracellularly it is able to serve as a potential late mediator of lethality. In the present study, we explored inflammation-promoting activity of HMGB1 and blockade of extracellular release of HMGB1 by glycyrrhizin (GL) in LPS/GalN-triggered mouse liver injury. At 1 to 10 h after LPS/GalN-treatment, mice were anesthetized to collect blood from heart puncture, and serum transaminase and HMGB1 were evaluated. Administration of LPS/GalN precipitated tissue injury associated with time-dependent alteration in HMGB1 serum levels. At 8 h nuclear immunoreactive products were remarkably reduced and extracellular HMGB1 expression was found exclusively in the pericentral foci. The treatment with GL significantly down-regulated the serum levels of ALT, AST, and HMGB1 in addition to the strong inhibition of tissue injury and extracellular immunoreactivity to HMGB1 and to acetylated-lysine. Furthermore, GL brought about a significant decrease in the number of apoptotic hepatocytes labeled with TUNEL-method. On the basis of these results, three apoptosis-associated genes were identified with microarray analysis and real-time PCR. The ChIP-assay revealed the binding of HMGB1 protein to *Gsto1* promoter sequence in LPS/GalN-treated mice and the remarkable decrease in combined HMGB1 protein by GL. The current findings claim that a single injection of LPS/GalN might stimulate apoptosis of hepatocytes through the binding of HMGB1 protein to *Gsto1* promoter region and that GL-treatment might prevent the apoptosis and inflammatory infiltrates caused with LPS/GalN-injection by disturbing the binding of HMGB1 protein to *Gsto1* promoter sequence.

## Introduction

High-mobility group proteins (HMGBs) are small DNA-binding proteins that serve an important role in transcriptional regulation [1]. One of these proteins, HMGB1 (amphoterin), has been identified as a late-acting mediator of lipopolysaccharide (LPS)-induced or sepsis-induced lethality in mice [2]. In addition to the role of a non-histone nuclear protein, HMGB1 also functions as an inflammatory cytokine when passively released from necrotic cells [3] or actively secreted from stress-received cells such as monocytes/macrophages in response to endotoxin, tumor necrosis factor (TNF)-α, or interleukin (IL)-1β [2], [4]–[6]. Once released into the intravascular space, HMGB1 can potentially amplify local inflammatory responses by enhancing the release of cytokines and chemokines from stressed cells [7] and interact with endothelial cells by up-regulating surface receptors and causing the secretion of soluble pro-inflammatory mediators [8]. Extracellular HMGB1 functions as a damage-associated molecular patterns (DAMPs) molecule and activates pro-inflammatory signaling pathways by enhancing pattern recognition receptors including toll-like receptor 4 (TLR4) and the receptor for advanced glycation end-products (RAGE) [5], [9]. Mounting evidence suggests that HMGB1 may also function to facilitate the recognition of other immune co-activators such as LPS, DNA, and IL-1 through greedy binding to these molecules [10]–[12].

To examine hepatic protection of some compound, acute hepatic injury induced by an intravenous injection of combination with a small dose of lipopolysaccharide (LPS) and D-galactosamine (GalN) has been widely used as an animal model [13] since the hepatic lesion in this model resembles that of human hepatitis [14]. We have reported that upon stimulation by LPS activated macrophages secrete various pro-inflammatory cytokines including IL-6, IL-10, IL-12 and TNF-α [15]. Among them, TNF-α is a key mediator causing hepatic apoptosis and necrosis in LPS/GalN-induced liver failure [16]. The number of apoptotic cells and the levels in serum concentration of TNF-α, IL-6, IL-10 and IL-12 as well as alanine aminotransferase (ALT) significantly increase after administration of LPS/GalN. However, glycyrrhizin (GL) has no effect on the production of these cytokines whereas it inhibits a significant increase in ALT levels and apoptotic cell numbers [15], [17].

GL is a biological active substance extracted from the licorice root (*Glycyrrhiza spp.*), which has been used for a folk medicine, and consists of one molecule of glycyrrhetinic acid and two glucuronic acids. Various pharmacological effects of GL are well known, such as anti-inflammatory [18], anti-viral [19], anti-allergic activities [20], hepatocyte proliferation [21] and hepatoprotection [22], [23]. Intravenous administration of GL improves ALT level of patients with chronic hepatitis. Especially in Japan, Stronger Neo Minophagen C (SNMC) has been used to remedy patients with hepatitis C [24], [25], and GL is main compound of SNMC. The effects and safety of SNMC have been assessed in Europe, too [26]–[29]. In addition, the research using nuclear magnetic resonance (NMR) and fluorescence methods reported the additional mechanism of GL that bound directly to HMGB1 and inhibited HMGB1 chemoattractant and mitogenic activities [30]. According to recent studies, furthermore, GL inhibits the cell proliferation and migration stimulated by HMGB1 as well as HMGB1-induced formation of blood vessels, and reduces inflammatory infiltrates [31].

Previous findings demonstrate that oxidative stress in hepatocytes leads to early shuttling of HMGB1 from the nucleus to cytoplasm, attended with its subsequent release in the absence of cell death [5], although it can be passively released following necrosis. The pathways governing HMGB1 release from hepatocytes are known to involve TLR4 activation and calcium signaling through calcium/calmodulin-dependent protein kinases (CaMKs) [5]. In other cell types, downstream events governing HMGB1 release have been linked to oxidation/reduction [32] and post-translational modifications that include phosphorylation [33] and acetylation [34]. However, in LPS/GalN-impaired liver it is unknown if post-translational modifications of HMGB1 regulates its release from hepatocytes. As HMGB1 is released from both stressed and necrotic cells, it might be useful to characterize the cellular expression and bloodstream kinetics of HMGB1 during LPS/GalN-induced liver injury. The purpose of this study is to investigate the post-translational pathway that regulates nuclear shuttling of HMGB1 into the cytoplasm and its subsequent release in the liver intoxicated with LPS/GalN. Furthermore, we aimed to evaluate the effect of GL as an inhibitor of HMGB1 upon the expanded kinetics of experimental hepatitis in mice treated with LPS/GaIN.

## Materials and Methods

### Animals and Materials

Male mice of the BALB/c strain weighing 23–25 g and aged 6 weeks were purchased from Japan SLC (Hamamatsu, Japan). The animals were maintained in an environmentally controlled room (24±1°C, 55±10% humidity) with alternatively 12-hour light-dark cycles. All experiments were approved by the Ethical Committee of Animal Experiments of the Tsurumi University School of Dental Medicine (Permit Number: 11085) and conformed to the guidelines established in the Guide for Care and Use of Experimental Animals, which are in compliance with the Nation Act on Welfare and Management of Animals. Lipopolysaccharide (LPS; *Escherichia coli*, O55:B5) and D-galactosamine (GalN) were purchased from Sigma-Aldrich (St. Louis, MO). All drugs were dissolved with pyrogen-free saline. Glycyrrhizin (GL) was obtained from Medical Chemistry Research Department of Minophagen Pharmaceutical Co., LTD. The BALB/c mice were intravenously injected with 25 ng LPS and 20 mg GalN per mouse. At 1 to 10 h after LPS/GalN-treatment, mice were anesthetized with intra-peritoneal injection of a mixture of 0.15 mg/kg medetomidine (Domitor, Zenyaku Co., Ltd., Tokyo, Japan), 2.0 mg/kg midazolam (Midazolam, SANDOZ, Tokyo, Japan), and 2.5 mg/kg butorphanol (Vetorphale, Meiji Seika Pharma Co., Ltd., Tokyo, Japan) to collect blood by cardiac puncture. Intraperitoneal administration of GL (50 mg/kg body weight) was performed 30 min before LPS/GalN-treatment. Control mice received equal volume of saline. Effects of GL on liver damage were examined 8 h after LPS/GalN-treatment.

### Analysis of Liver Enzymes

Hepatocellular damage was assessed by measuring serum alanine aminotransferase (ALT) and aspartate aminotransferase (AST) using a SPOT CHEM SP-4420 analyzer (ARKRAY, Kyoto, Japan).

### HMGB1 Enzyme-Linked Immunosorbent Assay (ELISA)

Serum HMGB1 was measured by ELISA using commercially available kits (HMGB1 ELISA Kit II; Shino-test, Sagamihara, Japan) following manufacturers' instructions. The sensitivity of the assay was 1.0 ng/mL.

### General Histology and Immunohistochemistry

At each stage from 1 to 10 h after treatment, mice were sacrificed under narcosis after collecting blood from heart puncture, the entire liver was quickly removed, and a tissue block was dissected out in the thickness of 1 mm. The specimens fixed with 0.1 M phosphate buffered (pH 7.4) 4% paraformaldehyde were embedded in paraffin wax after dehydration in a graded ethanol series. Some sections (6-μm in thickness) were stained with hematoxylin and eosin (HE).

For immunohistochemical identification of high-mobility group box 1 protein (HMGB1), lysine acetylation, and NF-κB p65, the streptavidin-biotin-peroxidase complex (SAB) method was applied [35]. In addition, we performed heat mediated antigen retrieval via the pressure cooker before commencing with immunohistochemical staining protocol for NF-κB p65. Specific cellular populations were stained with rabbit polyclonal antibodies to HMGB1 (1∶100–1∶400; ab18256, Abcam lnc, Cambridge, UK), to acetylated lysine (1∶50–1∶100; ab61257, Abcam), or to NF-κB p65 (1∶1000; ab7970, Abcam). For localizing the HMGB1 antigen to distinct cell types, double immunofluorescence staining was performed using the following antibodies: rabbit polyclonal antibodies for HMGB1 (1∶100) and rat monoclonal antibody for F4/80 (1∶100; ab6640, Abcam) that recognized a cytoplasmic antigen in monocyte-derived macrophages and Kupffer cells [36], [37], or rabbit HMGB1-antibody and hamster monoclonal anti-mouse CD11c (1∶100; ab33483, Abcam). As the secondary antibodies for double-immunofluorescence labeling, Alexa Fluor 488-labeled (green) goat anti-rat IgG (Invitrogen) for anti-F4/80 antibody, Alexa Fluor 488-labeled (green) goat anti-hamster IgG (Invitrogen) for anti-CD11c antibody, and Alexa Fluor 594-conjugated (red) goat anti-rabbit IgG (Invitrogen) for anti- HMGB1 antibody were used and counterstained with 4′,6-diamidine-2-phenylindole (DAPI; Roche Diagnostics, Basel, Switzerland). Stained sections were mounted with crystal/mount (Biomeda, Foster ctiy, CA) and observed under a fluorescence microscope (BZ9000-Analyzer, Keyence, Tokyo, Japan).

Two types of immunohistochemical controls were used. (1) Sections were processed by replacing the primary antibody with normal non-immune serum. (2) Sections were processed by replacing the primary antibody with an irrelevant antibody (GFP; Invitrogen). The immunoreactions were completely absent in control sections.

### Assessment of the Number of Hepatocytes Labeled with HMGB1 or Acetylated-Lysine

To evaluate the expression grade of HMGB1 or HMGB1 acetylation in hepatocytes, HMGB1- or acetylated-lysine-positive stained nuclei (HMGB1^+^ or acetylated-lysine^+^ hepatocytes) were counted in two high-power fields of x 400 per mouse at each stage, and the results were expressed as a percentage of total number of hepatocute nuclei. We randomly selected high-power fields of the pericentral region in control, LPS/GalN-, and GL+ LPS/GalN-treated groups (n = 4–6 mice per stage). The pericentral region was defined as the zone located within 120 μm from the walls of central veins [17].

### Isolation of Nuclear and Cytoplasmic Proteins

About 20 mg of frozen liver tissues were cut into small pieces using scalpel and placed into 2 mL microcentrifuge tubes. Qproteome Cell Compartment Kit (Qiagen, Hilden, Germany) was used according to the manufactur's protocol. Nuclear and cytosolic proteins were, respectively, collected and stored at −80°C. Protein concentration was determined with Pierce BCA protein assay reagent kit (Takara Bio, Inc., Shiga, Japan).

### HDAC colorimetric Activity Assay

HDAC Activity Colorimetric Assay kit (ab1432, Abcam) was used according to the manufactur's protocol. Briefly, 100 μg of nuclear or cytosolic protein was diluted in a final volume of 85 μL of ddH_2_O and placed in a round bottom 96-well plate. 10 μL of the 10× assay buffer was added to each cell followed by 5 μL of the HDAC colorimetric substrate. The mixture was incubated at 37 °C for 3 h. 10 μL of Lysine Developer was then added, and the plate was incubated at 37 °C for 30 min. The sample was read in an ELISA plate reader at 405 nm.

### TUNEL staining

The nuclear DNA fragmentation of apoptotic cells was labeled *in situ* by the terminal deoxynucleotidyl transferase-mediated dUTP nick end labeling (TUNEL) method [17]. After treating with 20 mg/mL proteinase K (Roche Diagnostics), the sections were incubated with 0.3 U/mL terminal deoxynucleotidyl transferase (Invitrogen) and 0.04 nM biotinylated dUTP (Roche Diagnostics) in terminal deoxynucleotidyl transferase buffer (Invitrogen) at 37°C. After rinsing, the sections were incubated with peroxidase-conjugated streptavidin (DAKO) diluted 1∶300 in PBS and peroxidase activity was visualized with 0.05% DAB (Sigma-Aldrich) containing 0.01% CoCl_2_ and 0.01% H_2_O_2_.

### Quantification of TUNEL-positive cells

We counted TUNEL-positive cells distributed in the pericentral region (see above-mentioned item) in control, LPS/GalN-, and GL+LPS/GalN-treated groups on clearly stained paraffin sections at the light-microscopic level. Four to five mouse livers per group were examined and the number of labeled cells was determined in one to six fields per liver. The size of the area was measured by use of an Image Processor and Analyzer (TRI/2 D-MES; RATOC, Tokyo) and cellular density was expressed as the cell number per square millimeter.

### Gene microarray analysis

RNA extraction and amplification: Sections (6 μm in thickness) were prepared from each paraffin-embedded specimen fixed with buffered-paraformaldehyde. After deparaffinization, tissues were treated with proteinase K at 37°C overnight. Following centrifugation, the supernatant was processed with a silica-based spin column (Toray Industries, Inc., Tokyo, Japan) in order to obtain purified total RNA. The degrees of RNA cross-linking and RNA degradation were analyzed by electrophoresis using an Agilent 2100 Bioanalyzer (Agilent Technologies, Santa Clara, CA). 1 mg of each total RNA was amplified using 3D-Gene FFPE Gene Expression Analysis Reagent kit (Toray industries, Inc., during preparations for launch) in accordance with manufacturer's instructions. The amplified anti-sense RNAs (aRNAs) were labeled with Cyanine 5 (Cy5, GE Healthcare) and were subjected to hybridization.

Hybridization: 1 mg of each Cy5-labeled RNA solution was applied to 3D-Gene Mouse Oligo chip 24 k (Toray Industries, Inc., 23,522 distinct genes) and hybridized for 16 h according with manufacturer's instructions. The hybridization signals were obtained using 3D-Gene Scanner (Toray Industries Inc.) and processed by 3D-Gene Extraction (Toray Industries Inc.). The signals for each gene were normalized using a global normalization method (median Cy3/Cy5 ratio  = 1).

### Quantitative Analysis of Gene Expression Using Real Time PCR

For semiquantitative analysis of *Rage*, *toll-like receptor 4* (*Tlr4*), *Pdcd6, Gsto1*, *Cyp2d10*, and *Stc1* mRNA, real-time PCR was performed with the control, LPS/GalN, and GL+ LPS/GalN groups. After animals were sacrificed and their livers were separated, total RNA of the sample was extracted using TRIZOL (Invitrogen) and made into cDNA with SuperScript III First-Strand Synthesis System for RT-PCR (Invitrogen) according to the manufacturer's instructions. Primer sequences of *Rage*, *Tlr4, Pdcd6, Gsto1*, *Cyp2d10*, and *Stc1* are described as follows (forward, reverse): *Rage*; 5′-ACATACAAGATGGACCTGTGGA-3′, 5′-CCAGTTCATTCACACCAGGA-3′, *Tlr4*; 5′-GGACTCTGATCATGGCACTG-3′, 5′-CTGATCCATGCATTGGTAGGT-3′, *Pdcd6*; 5′-AGAAGTTTGGGGAAGAGATCG-3′, 5′-CGAGAGGCCACATTCTTGAT -3′, *Gsto1*; 5′-CAGCGATGTCGGGAGAAT-3′, 5′-GGCAGAACCTCATGCTGTAGA-3′, *Cyp2d10*; 5′-GCAGAAAGTACTGGAAGATAGTTTGA-3′, 5′-AGGAGTATGGGGAACATATTAAGAAC-3′, *Stc1*; 5′-GAGGCGGAACAAAATGATTC-3′, 5′-GCAGCGAACCACTTCAGC-3′. As probe for *Rage, Tlr4*, *Pdcd6, Gsto1*, *Cyp2d10*, and *Stc1*, probe #71, #2, #84, #60, #106, and #45 (Universal probe library; Roche Diagnostics) were respectively used and Fast Start Universal Probe Master (Roche Diagnostics) as PCR reagent. PCR reaction was observed using Applied Biosystems 7900HT Fast Real-Time PCR systems (Applied Biosystems, Carlsbad, CA) and PCR condition was as follows: 50°C 2 min, 95°C 10 min, 50 cycles at 95°C 15 seconds, 60°C 1 min. *Actb* (*β-Actin*) was used as an internal control (Universal Probe Library Mouse ACTB Gene Assay; Roche Diagnostics).

### Chromatin immunoprecipitation (ChIP)

ChIP assay was conducted using the EpiQuick Tissue Chromatin Immunoprecipitation Kit (Epigentek, Brooklyn, NY) according to the manufacture's instruction and rabbit polyclonal antibody against HMGB1 (ab18256, Abcam). The immunoprecipitated chromatin was analyzed in duplicate by PCR using the primers (Mo-Gsto1-ChIP-2-F: 5′-AAAGTGGACGAAACCCTTGA-3′ and Mo-Gsto1-ChIP-2-R: 5′-CACACACGCATGTGACAGAA-3′) for mouse *Gsto1* promoter. The binding site was identified using Transcription Factor Search web site (http://www.cbrc.jp/research/db/TFSEARCH.html). After cloned the PCR products into pCR2.1 vector using TA cloning kit (invitrogen), the sequence of PCR products was confirmed.

### Statistical Analysis

All Data were showed as the mean ± standard error of mean (SEM). The Grubbs and Smirnov methods were used to exclude outliers, and statistical significance of differences between the LPS/GalN-treated group and test group was evaluated by a t-test with pooled standard division and finally was corrected with *p* value adjustment from the Bonferroni method. Differences were considered to be significant for *p* values less than 0.05.

## Results

### Time-Dependent Changes of Serum ALT, AST, and HMGB1 Levels in Mice Treated with LPS/GalN

**Figure 1:**
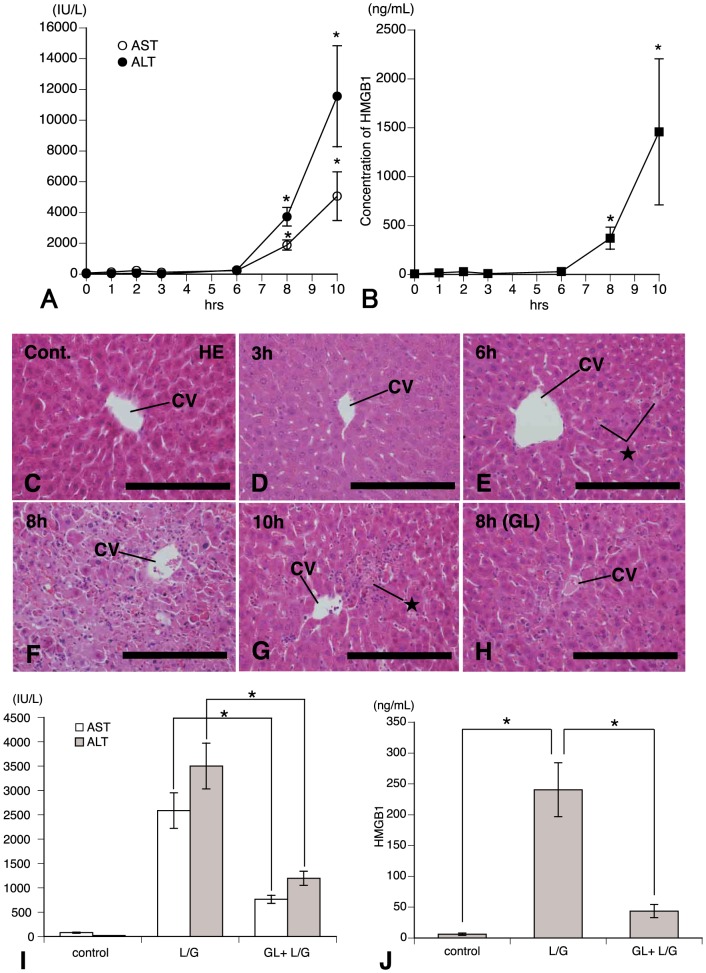
Time-dependent changes of serum ALT, AST, and HMGB1 levels in LPS/GalN-treated mice. Serum levels of ALT, AST (**A**) and HMGB1 (**B**) at various times after LPS/GalN-treatment. (**C**–**H**) HE-stained paraffin sections showing the histopathological conditions of control (**C**), 3 (**D**), 6 (**E**), 8 (**F**), and 10 h (**G**) after LPS/GalN-treatment. In addition, HE-stained section (**H**) presents inhibitory effect of glycyrrhizin (GL) on liver injury. At 6 (**E**) and 10 h (**G**) some injured hepatocytes (*★*) are found within the hepatic lobuli and strong tissue injury is observed in the pericentral area at 8 h. *CV*: central vein. *Bars*  = 200 μm for **C**–**H**. GL inhibits LPS/GalN-induced increase in serum ALT, AST (**I**), and HMGB1 (**J**) levels measured at 8 h after treatment. Each value represents the mean±SEM of 6 animals. *Significant difference from values of control (P<0.05) or between groups treated with LPS/GalN alone and GL plus LPS/GalN (P<0.05).

To test if engagement of HMGB1 mediated hepatic injury in mice treated with LPS/GalN, we measured the levels of serum ALT, AST, and HMGB1 at different time points. The intravenous administration of LPS/GalN in the mice stimulated their liver inflammation associated with alteration in HMGB1 levels. Serum ALT and AST levels did not show a significant increase for up to 6 h after LPS/GalN-treatment (Fig.1A). In contrast, at 8 h to 10 h significant increases in serum levels of ALT and AST were detected in LPS/GalN-treatment versus control (Fig.1A). An elevation in serum concentrations of HMGB1, as well as ALT and AST, was significantly greater by 10 h (Fig. 1B). Thereafter, increased ALT, AST, and HMGB1 levels were reduced with time after treatment (data not shown). A coefficient of correlation between ALT and HMGB1 was 0.95 and the both levels showed strongly mutual relation. These findings suggest that HMGB1 contributed to hepatic injury in mice treated with LPS/GalN and led us to dissect the contribution of HMGB1 to these processes.

**Figure 2:**
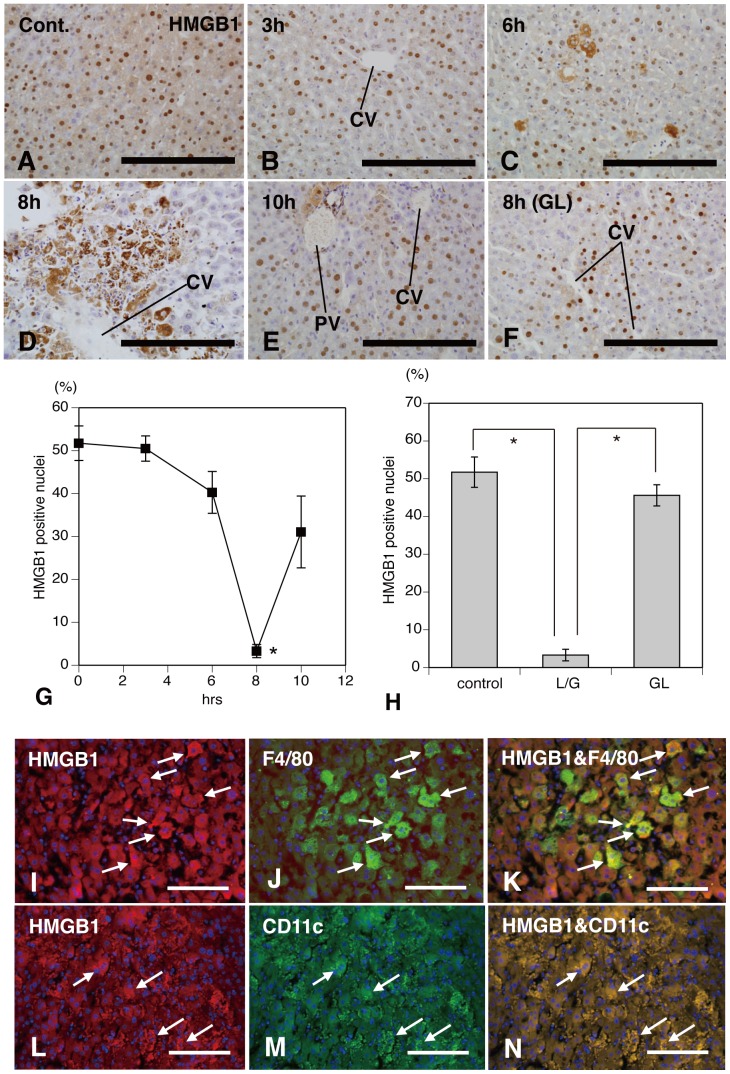
Translocation of HMGB1 into extranuclear and extracellular milieu in LPS/GalN-treatment. Immunohistochemical staining for HMGB1 shows a distinct nuclear expression in the hepatocytes of control mice (**A**) and 3 h after treatment (**B**). The imunoreactive products are reduced in the nuclei of hepatocytes at 6 h (**C**), and extracellular expression (**D**) is intensely labeled in the pericentral areas as well as suppressive intranuclear immunoreactions (**G**) at 8 h. At 10 h a distinct nuclear expression for HMGB1 is significantly increased in the hepatocytes compared with 8 h after treatment (**E**, **G**). GL-treatment strongly inhibits immunoreactivity of cytoplasmic and additional extracellular HMGB1 (**F**) and significantly retrieve a decrease of intranuclear immunoreactive products (**H**). Numerous HMGB1- (*white arrows* in **I** and **L**), F4/80- (*white arrows* in **J**), or CD11c-positive cells (*white arrows* in **M**) are distributed in the inflammatory pericentral areas at 8 h after LPS/GalN treatment. Some of HMGB1-positive cells are simultaneously stained with antibodies to F4/80 (*white arrows* in figure **K**) or to CD11c (*white arrows* in figure **N**). *CV*: central vein; *PV* portal vein; *Bars*  = 200 μm for **A**–**F**, 100 μm for **I**–**N**. *Significant difference between the values of 8 h after LPS/GalN-treatment and of other hours after treatment in figure **G** (*P*<0.05). *Significant difference from values of control or of the group treated with GL plus LPS/GalN in figure **H** (*P*<0.05).

**Figure 3:**
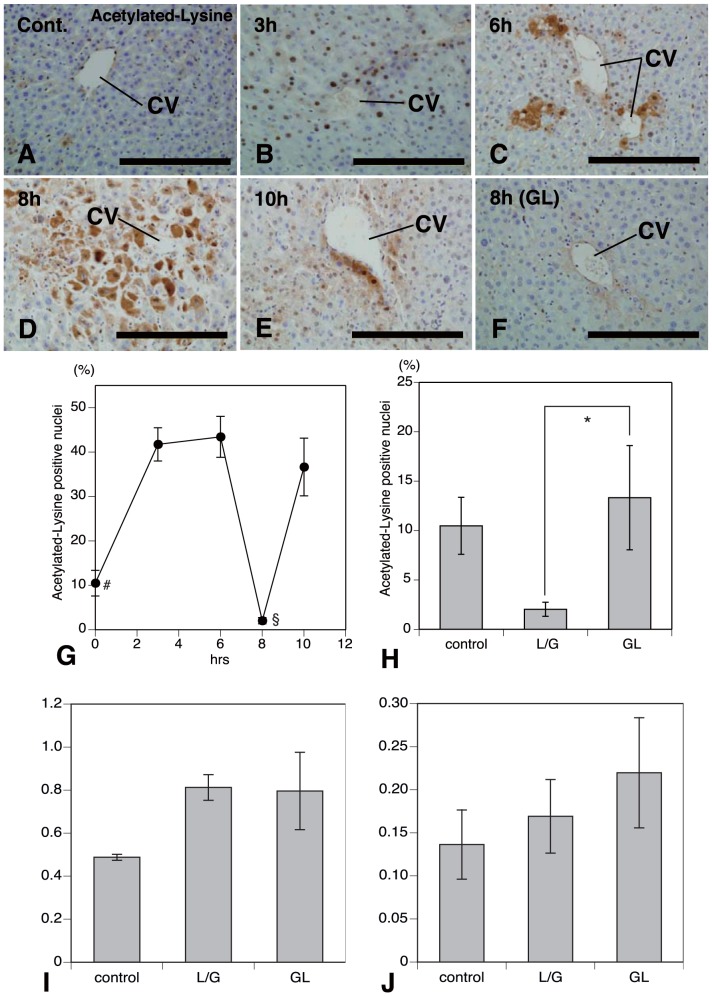
Acetylation of HMGB1 and HDAC activity by LPS/GalN-treatment. Immunohistochemistry with antibodies against acetylated-lysine (**A**–**H**) and HDAC activity (**I**, **J**). Immunoreactive products are found in the nuclei of only a few hepatocytes in control mice (**A**, **G**). The hepatocytes with nuclear immunoreactivity are significantly increased in number 1–6 h after treatment (**B**, **C**, **G**). At 8 h of the concomitant administration of LPS/GalN, the nuclear immunoreactivity for acetylated-lysine is strongly reduced (**D**, **G**) and immunolabeled products are exclusively found in the extracelluar milieu of pericentral areas (**D**). At 10 h a distinct nuclear expression of acetylated-lysine is significantly increased in hepatocytes (**E**, **G**). Administration of GL remarkably reduces the immunoreactive, cytoplasmic and extracellular response to acetylated-lysine in the pericentral areas (**F**) and shows a significant increase in the number of nuclear immunoreactive products for acetylated-lysine (**H**). Colorimetric assay for histone deacetylase (HDAC) activity shows that the cytoplasmic (**I**) and nuclear (**J**) HDAC activity is increased in both liver tissues after LPS/GalN- or GL+LPS/GaIN-treatment compared with baseline animals, but not significant. *CV*: central vein; *Bars*  = 200 μm for **A**–**F**. *#*Significant difference from values of 3 h after LPS/GalN treatment (*P*<0.05; **G**), *§*significant difference from values of 6 or 10 h after treatment (*P*<0.05; **G**), and *significant difference between groups treated with LPS/GalN alone and GL plus LPS/GalN (*P*<0.05; **H**).

Histological examination of livers from LPS/GalN-treated mice revealed signs of inflammatory liver damage (Fig. 1C–G). On the hematoxylin and eosin (HE)-stained sections, the signs of acute liver injury and damage to hapatocytes were not found in mice of the control (Fig. 1C) and 1–3 h after treatment (Fig. 1D). At 6 h injured hepatocytes were scatteredly distributed within the lobules (Fig. 1E). Numerous inflammatory foci were observed in the pericentral area, consisting of infiltrating cells including neutrophils or monocytes/macrophages and injured hepatocytes after 8 h of treatment (Fig. 1F). On the contrary, these injured foci were considerably reduced at 10 h (Fig. 1G), whereas an elevation in serum concentrations of ALT, AST, and HMGB1 continued by this time (Fig. 1A, B). GL-treatment performed 30 min before LPS/GalN injection strongly inhibited both inflammatory foci (Fig. 1H) and enhancement in serum levels of ALT, AST, and HMGB1 (Fig. 1I, J), induced at 8 h after injection of LPS/GalN.

### LPS/GalN-Treatment Induces the Translocation of HMGB1 into Extranuclear and Extracellular Milieu

To determine the cellular localization of HMGB1 in the remnant after treatment, immunohistochemical staining was performed on paraffin sections retrieved from mice at various hours after LPS/GalN treatment (Fig. 2A–E). Immunohistochemistry with antibodies against HMGB1 revealed a distinct nuclear expression in the hepatocytes of control mice (Fig. 2A) and 1–3 h after treatment (Fig. 2B). At these stages, some cells expressed the weak cytoplasmic immunoreaction for HMGB1, but no deposition of HMGB1 was seen in the extracellular milieu. Immunoreactions for HMGB1 in the nuclei began to be suppressed 6 h after the concomitant administration of LPS/GalN (Fig. 2C), and at 8 h intranuclear immunoreactive products were remarkably reduced (Fig. 2G) and aberrant, extracellular expression of HMGB1 was demonstrated (Fig. 2D). At this stage, immunolabeled HMGB1 was restricted to inflammatory pericentral areas. Pronounced cytoplasmic and additional extracellular HMGB1 expression appeared to coincide with the progression of inflamed liver (Figs. 1, 2). At 10 h a distinct nuclear expression for HMGB1 was again significantly increased in the hepatocytes (Fig. 2G), although occational hepatocytes remained to express cytoplasmic HMGB1 (Fig. 2E). GL-treatment strongly inhibited immunoreactive expression of cytoplasmic and additional extracellular HMGB1 (Fig. 2F) and a decrease in intranuclear immunoreactive products (Fig. 2H), induced by injection of LPS/GalN.

### Cellular Expression of HMGB1 in Activated Kupffer and Dendritic Cells

We examined the immunoreactive expression of HMGB1 by activated Kupffer and dendritic cells in response to LPS/GalN-induced hepatic injury using double-immunofluorescence staining for HMGB1/F4/80 (Fig. 2I–K) or HMGB1/CD11c (Fig. 2L–N). Some cell populations distributed in the inflammatory pericentral foci were intensely labeled for HMGB1 (*white arrows* in Fig. 2I, L), F4/80 (*white arrows* in Fig. 2J), or CD11c (*white arrows* in Fig. 2M) 8 h after LPS/GalN-treatment. Furthermore, a part of F4/80^+^ Kupffer and CD11c^+^ dendritic cells were simultaneously immunolabeled for HMGB1 (*white arrows* in Fig. 2K, N), respectively. These results indicate that activated Kupffer and dendritic cells expressed HMGB1 in their cytoplasm during the LPS/GalN-induced hepatic injury.

### Acetylation of HMGB1 in the Liver Injury Induced with LPS/GalN

Acetylation is a post-translational modification that can influence protein localization and function. Hyperacetylation shifts the equilibrium of HMGB1 from a predominant nuclear location toward cytosolic accumulation and leads to subsequent release [34], [38]. To determine the acetylation status of HMGB1in mouse liver subjected to the concomitant administration of LPS/GalN, the levels of acetylated HMGB1 were assessed by immunohistochemistry with polyclonal antibodies against acetylated-lysine (Fig. 3A–H). A few nuclear immunoreactive products were labeled in the hepatocytes of control mice (Fig. 3A, G). The liver cells with nuclear immunoreactivity for acetylated-lysine were significantly increased in number 1–3 h after treatment (Fig. 3B, G) and some cells expressed the weak cytoplasmic immunoreaction for acetylated-lysine, but no deposition of acetylated-lysine was seen in the extracellular milieu. After 6 h of the concomitant administration of LPS/GalN, the immunoreactive intensity for acetylated-lysine in the nuclei began to be suppressed and immunolabeled products were extracellularly stained in the pericentral areas (Fig. 3C). Intranuclear immunoreactive products were remarkably reduced at 8 h (Fig. 3D, G) and aberrant, extracellular acetylated-lysine expression was frequently found (Fig. 3D). At this stage, immunolabeled acetylated-lysine was exclusively restricted to the inflammatory pericentral areas. Pronounced cytoplasmic and additional extracellular acetylated-lysine expression seems to coincide with both the progression of inflammation in the liver (compare Fig. 3 with Fig. 1) and time-dependent change in the distributional pattern of immunolabeled HMGB1 (compare Fig. 3 with Fig. 2). At 10 h a distinct nuclear expression of acetylated-lysine was significantly increased in the liver cells (Fig. 3G), although some liver cells expressed cytoplasmic acetylated-lysine in the pericental areas (Fig. 3E). In the liver of mice treated with LPS/GalN, administration of GL remarkably reduced the immunoreactive, cytoplasmic and extracellular response to acetylated-lysine (Fig. 3F) and showed a significant increase in number of nuclear immunoreactive products for acetylated-lysine (Fig. 3H). These data suggest that the treatment with GL suppressed the translocation of HMGB1 into the extracellular milieu through the blockade of acetylation of HMGB1 and inhibited rapid activation of pro-inflammatory pathways accompanying the LPS-GalN-induced liver injury. However, mechanisms governing the translocation of HMGB1 recognized in the injured liver induced by an injection of LPS/GalN remain to be unknown.

### Nuclear HDAC Activity in the LPS/GalN-Induced Liver Injury

As LPS/GalN-treatment caused a temporary increase in the number of hepatocytes with acetylated-lysine^+^ nucleus (Fig. 3G), we conducted colorimetric assays to assess nuclear and cytoplasmic histone deacetylase (HDAC) activity during LPS/GalN-induced liver injury. The cytoplasmic (Fig. 3I) and nuclear (Fig. 3J) HDAC activity was increased in both liver tissues from animals subjected to LPS/GalN- or GL+LPS/GaIN-treatment compared with baseline animals, but not significant. Thus, the current data do not support the past working hypothesis [38] that a decrease in nuclear or cytoplasmic HDAC activity due to LPS/GalN-treatment shifts the equilibrium of HMGB1 from a predominant nuclear location toward cytosolic accumulation, leads to subsequent release, and induces hepatic injury.

### Association of HMGB1 with TLR4 or RAGE in Mice Treated with LPS/GalN

**Figure 4:**
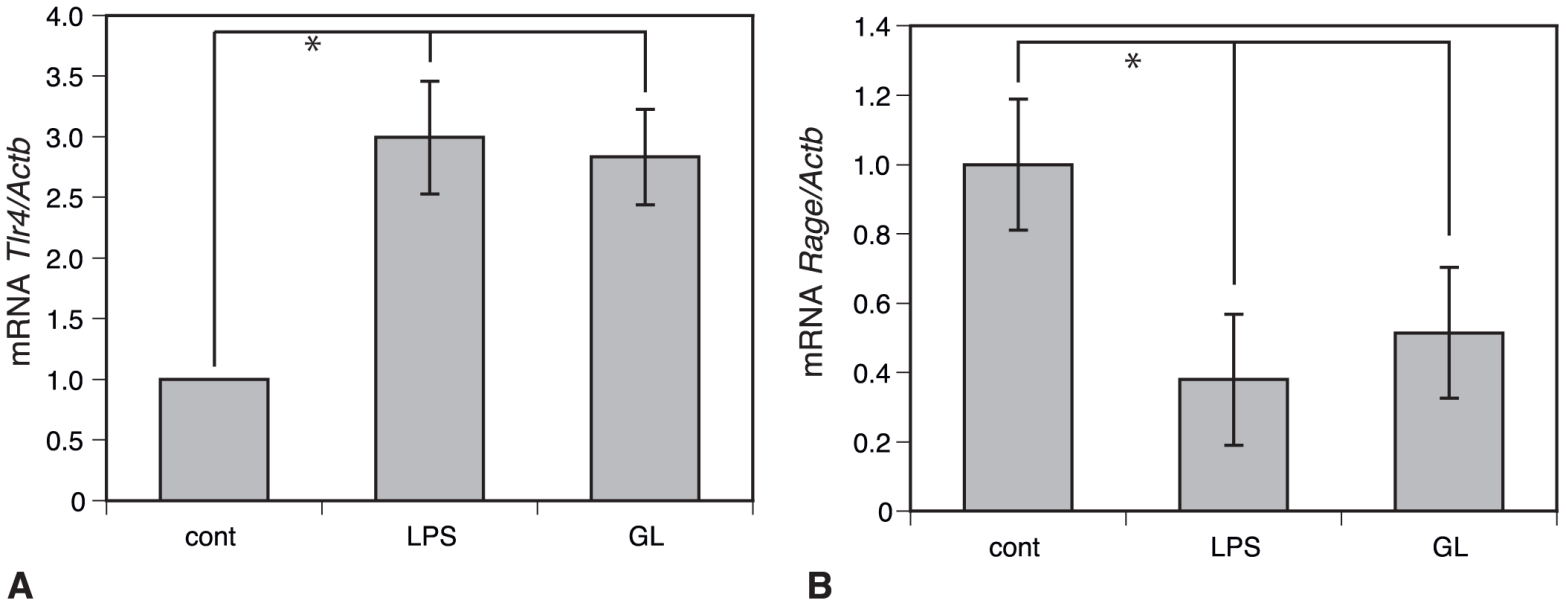
Expression pattern of *Tlr4* and *Rage* mRNA in mice treated with LPS/GalN. Real time PCR in mRNA levels of *Tlr4* and *Rage*. LPS/GalN-treatment significantly stimulates an increase in mRNA levels of *Tlr4* (**A**), whereas it induces a significant decrease in mRNA levels of *Rage* (**B**). Furthermore, an administration of GL does not become responsive to mRNA levels of both the *Tlr4* and *Rage* in mice intoxicated with LPS/GalN. *Significant difference from values of control (*P*<0.05).

TLR2, 4, and RAGE have been so far identified to be implicated in HMGB1 signaling [39]. To evaluate the involvement of TLR4 and RAGE as the receptor of HMGB1, we measured the kinetics in mRNA levels of these receptors induced by LPS/D-GalN-treatment using real time PCR (Fig. 4). LPS/GalN induced a significant decrease in mRNA levels of *Rage* (Fig. 4B), whereas it significantly stimulated an increase in mRNA of *Tlr4* (Fig. 4A). In addition, an administration of GL did not become responsive to both the *Tlr4* and *Rage* in mice treated with LPS/GalN (Fig. 4A, B). Consequently, our findings indicate that LPS/GalN-induced hepatic injury might not be caused by extracellularly released HMGB1 via receptors such as TLR4/RAGE.

### Apoptotic cells induced by LPS/GalN-treatment

**Figure 5:**
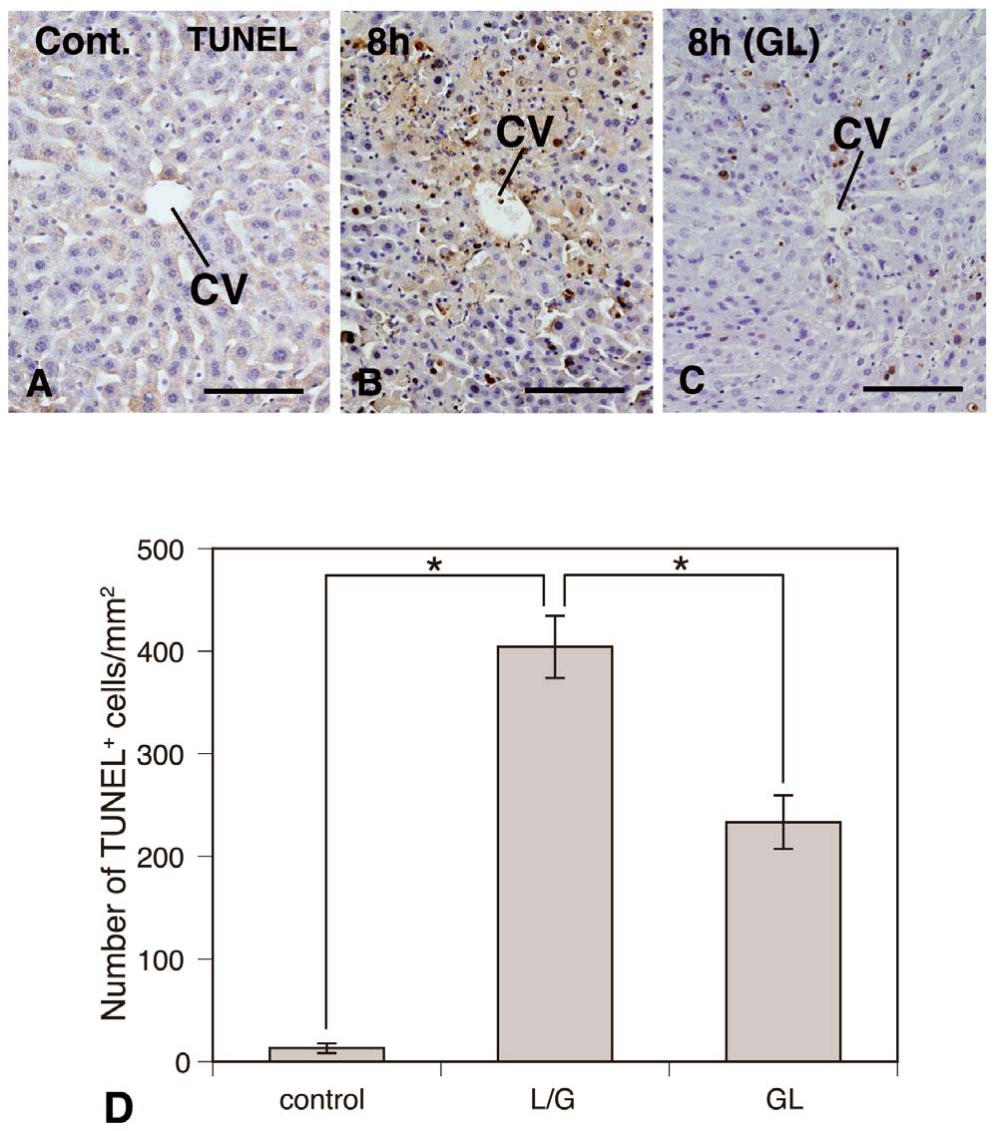
Apoptotic cell death induced by LPS/GalN-treatment. TUNEL-method showing apoptotic liver cells and their distributional patterns. Control (**A**), LPS/GalN-treatment (**B**), and GL+LPS/GalN-treatment (**C**). Intraperitoneal administration of GL significantly reduces the number of TUNEL-labeled cells in pericentral areas (**B**–**D**). *Significant difference between two groups (Control versus LPS/GalN or LPS/GalN versus GL+LPS/GalN) approved (*P*<0.05).

Apoptotic liver cells and their distributional patterns were examined using the TUNEL-method. As expected, few TUNEL-positive cells were observed in the liver of saline-injected control mice (Fig. 5A). On the contrary, numbers of TUNEL-positive cells with sharply delineated masses or crescents of condensed chromatin were identified in the inflammatory pericentral areas at 8 h post-injection of LPS/GalN (Fig. 5B). The number of TUNEL-positive cells was significantly increased in the pericentral areas of the LPS/GalN-treated liver compared with controls (Fig. 5D). Intraperitoneal administration of GL significantly reduced the number of TUNEL-labeled cells in pericentral areas compared with mice treated with LPS/D-GalN (Fig. 5B–D). From the current results, mouse liver injury intoxicated with LPS/GalN might be caused through the apoptosis of hepatocytes, which might be improved by the inhibition of apoptotic cell death due to an injection of GL.

We, next, explored the fluctuation in the expression of genes regulating apoptotic cell death in LPS/GaIN-injured liver using the gene microarray analysis.

### Injection of LPS/GalN Regulates the mRNA Expression of *Pdcd6, Gsto1, Cyp2d10*, and *Stc1* of Gene Family Involved in Apoptosis

**Figure 6:**
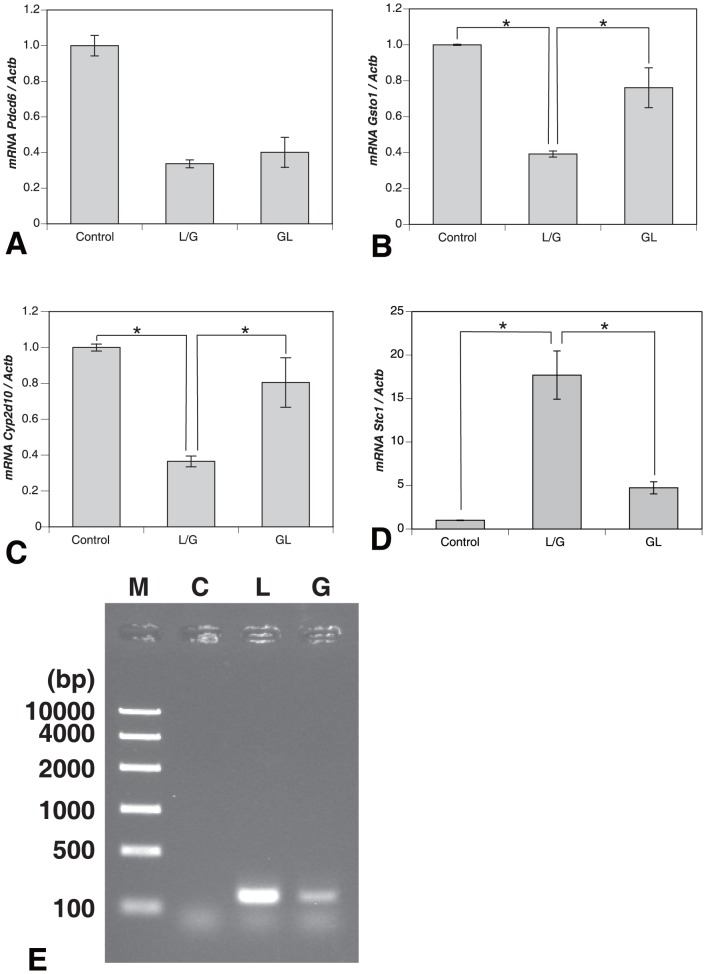
mRNA expression of gene family involved in apoptosis by LPS/GalN-treatment. PCR using the cDNA from control, LPS/GalN-injected, and GL+LPS/GalN-treated livers. The expression pattern of *Pdcd6* (**A**) is not influenced by LPS/GalN treatment, but that of 3 genes of *Gsto1* (**B**), *Cyp2d10* (**C**), and *Stc1* (**D**) by treatment. After an injection of LPS/GalN, the expression levels of *Gsto1* (**B**) *and Cyp2d10* (**C**) mRNAs are significantly decreased. The expression levels of *Gsto1 and Cyp2d10* mRNAs are significantly recovered by administration of GL. In contrast, the expression level of *Stc1* (**D**) mRNA is significantly increased in LPS/GalN-injected liver injury and that of *Stc1* mRNA is significantly down-regulated by administration of GL. ChIP assay analysis shows that HMGB1 protein intensely binds to *Gsto1* promoter sequence in LPS/GalN-induced liver injury (**E**), and an administration of GL remarkably inhibits the binding of Hmgb1 to *Gsto1* promoter sequence. *Significant difference between two groups (Control versus LPS/GalN or LPS/GalN versus GL+LPS/GalN) approved (*P*<0.05). *L/G*: LPS/GalN, *GL*: GL+LPS/GalN in **A**–**D**; *M*: marker, *C*: control, *L*: LPS/GalN, *G*: GL+LPS/GalN in **E**.

A microarray analysis revealed that the expression pattern of 4 genes, *Pdcd6*, *Gsto1*, *Cyp2d10*, and *Stc1*, was influenced by injecting LPS/GaIN or LPS/GalN with GL (Fig. S1). The expression levels of *Pdcd6*, *Gsto1*, and *Cyp2d10* were down-regulated in LPS/GalN-injected liver injury and recovered by injection of GL. On the contrary, *Stc1* expression pattern was up-regulated by LPS/GalN treatment and restored by injection of GL. To validate these results from microarray analysis, we performed semi-quantitative PCR using the cDNA from control, LPS/GalN-injured, and GL+LPS/GalN-treated livers. After an injection of LPS/GalN, the expression levels of *Gsto1 and Cyp2d10* mRNAs were significantly decreased to 0.392±0.017 and 0.365±0.030, respectively. The expression levels of *Gsto1 and Cyp2d10* mRNAs were significantly recovered to 0.761±0.111 and 0.805±0.138, respectively, by administration of GL (Fig. 6B, C). Also, the expression level of *Stc1* mRNA was significantly increased to 17.697±2.775 in LPS/GalN-injected liver injury and that of *Stc1* mRNA was significantly down-regulated to 4.734±0.695 by administration of GL (Fig.6D). On the other hand, the mRNA levels of *Pdcd6* were not significantly affected by LPS/GalN- or GL+LPS/GalN-treatment (Fig. 6A). These findings apparently show that LPS/GalN-induced hepatic injury in this animal model might be brought about through the apoptosis signaling. However, it remains to unknown whether HMGB1 is implicated in apoptosis signaling in the liver remnants treated with LPS/GalN.

### HMGB1 Protein Binds to *Gsto1* Promoter Sequence

To confirm the function of HMGB1 protein in this experimental model, chromatin immunoprecipitation (ChIP) assay was performed. Using Transcription Factor Search web site, we designed several respective PCR primers to amplify *Gsto1*, *Cyp2d10*, or *Stc1* promoter regions. We could amplify expected size of band, clone the PCR product into PCR2.1 vector, and confirm the sequence, only with the use of the primers to amplify *Gsto1* promoter region (−3437 to −3282 bp). Chip assay analysis revealed that HMGB1 protein strongly bound to *Gsto1* promoter sequence in LPS/GalN-injected liver injury (Fig. 6E), but we could not detect a combination with *Cyp2d10* or *Stc1* promoter sequence using this analysis (data not shown). Furthermore, an administration of GL remarkably inhibited the binding of HMGB1 to *Gsto1* promoter sequence (Fig. 6E).

## Discussion

Mouse liver injury induced by lipopolysaccharide/D-galactosamine (LPS/GalN) is commonly used as a promising animal model for elucidating the mechanism of clinical dysfunction and for evaluating the efficacy of hepatoprotectives [15], [17], [40]. Multiple pathways converge to signal activation of endogenous inflammatory cells within the liver, as well as upregulation of key adhesion molecules and chemokines that mediate migration of inflammatory cells from the periphery into foci of activation and inflammation in the perturbed remnant. Once set in motion, these facets of the immune-inflammatory response join forces to stimulate tissue-destructive pathways and failure of regenerative programs [41]–[44]. In this study, we provide the first *in vivo* evidence showing that HMGB1 is involved in the apoptosis of hepatocytes caused by LPS/GalN-treatment and administration of GL significantly improves hepatic injury, in parallel with suppression of exaggerated apoptotic cell death and enhanced expression of regeneratiom mediator. HMGB1 is a multifunctional protein: its earliest functions were described as a non-histone DNA-binding nuclear protein. HMGB1 binds to DNA in a sequence-independent manner and modifies DNA structure to facilitate transcription, replication, and repair [45], [46]. These functions are essential for survival, as HMGB1-deficient mice die of hypoglycemia within 24 hours after birth [47]. Recently, HMGB1 has been identified as a novel inflammatory cytokine and a late mediator of endotoxin lethality in mice [48]. Extracellular HMGB1 activates a large number of different physiological responses in different cell types [49]. HMGB1 may be released both through active secretion from various cells, including activated monocytes/macrophages [2], neutrophils [50], and apoptotic cells [51], and passively as a danger signal from necrotic cells [3]. Through the TLR4 system, HMGB1 produces an early inflammatory response [4], leading to amplification of HMGB1 secretion [5]. Active HMGB1 secretion from phagocytes displays delayed kinetics [2], [52], [53]. In the current experimental model, we could recognize the earliest biochemical and histological damage at 6 h stage after LPS/GalN-treatment. Thus, this delayed active HMGB1 release may partly explain some of our findings. The present immunohistochemistry revealed that the intense expression of HMGB1 focused on the inflammatory foci close to the central veins, that is, on the area susceptible to LPS/GalN-treatment. Although the immunoreactive products of HMGB1 reached a maximum by 8 h, levels of HMGB1 in the serum were maximal at 10 h. Increase in the serum levels of HMGB1 seems to occur a little later than pathological damages in liver tissue induced with LPS/GalN-treatment. Thus, the observed efflux of HMGB1 appears to derive mainly from injured hepatic tissues. These results are identical with data obtained from patients with acute liver failure [54]. During LPS/GalN-induced liver injury, HMGB1 kinetics is distinct from that of TNF-α, IL-6, IL-10 and IL-12 obtained in our previous study [15]. This is in line with other previous works failing to find an association between HMGB1 and various cytokines (TNF-α, IL-6, and IL-10) [6], [55] and might reflect the complexity of inflammatory response in the clinical setting.

The role of the receptor for advanced glycation end products (RAGE) and its ligands in maintaining and amplifying inflammation has been recently highlighted [56]. RAGE, a member of the immunoglobulin superfamily, interacts with ligands enriched in flamed milieu, includimg (carboxymethyl)lysine-modified adducts and S100/calgranulins, the latter members of a family of pro-inflammatory cytokines [57], [58]. Blockade of RAGE, employing soluble RAGE (sRAGE), the extracellular ligand-binding domain of RAGE, suppresses injury in hepatic ischemia/reperfusion [59], as well as the induction of collagen-induced arthritis in mice sensitized to and challenged with bovine type II collagen [60]. In this study, however, the expression levels of *Rage* mRNA were conversely decreased by administration of LPS/GalN and GL-treatment did not exert any influence on its expression. In contrast, the expression levels of *Tlr4* mRNA were significantly increased in LPS/GalN-induced hepatic injury as compared with the control, but GL did not inhibit it. GL binds directly to each of two HMG boxes of HMGB1, as shown by NMR and fluorescence studies [30]. The modest effect of GL on the intranuclear function of HMGB1 is agreement with the absence of cytotoxicity even at high GL concentrations and with the good pharmacological tolerability of GL in rodents and humans [61]. Interestingly, administration of GL does not cause the release of HMGB1 from apoptotic chromatin, implying that it will not produce paradoxical inflammatory responses to apoptotic cells [30]. The current results suggest that the effective mechanisms of GL are down-stream of initial TLR activation in the injured liver induced by LPS/GalN injection.

Inside the cell, HMGB1 binds DNA and regulates transcription [62], whreas outside the cell, it serves as a cytokine and mediates the late effects of LPS [2]. In the present study, the double-immunofluorescence analysis for HMGB1/F4/80 or HMGB1/CD11c revealed that activated macrophages and dendritic cells expressed HMGB1 in their cytoplasm in the LPS/GalN-induced hepatic injury, respectively. HMGB1 in monocytes and macrophages is extensively acetylated upon activation by LPS, causing localization of the protein to the cytosol [34]. Cytosolic HMGB1 is then concentrated into secretory lysosomes and secreted when the cells received an appropriate second signal [63]. The movement of HMGB1 into the extracellular space has been demonstrated for macrophages stimulated with LPS [34], [49], [64] as well as cells undergoing necrosis [3], [65] or apoptosis [51]. In our previous paper [17], analyses using TUNEL-method, an oligonucleosome-bound DNA ELISA, and microdissection-method showed that the degree of hepatic injury is associated with a substantial number of cells undergoing apoptosis in acute hepatitis induced with a single injection of LPS/GalN. GL-treatment suppressed the apoptosis of liver cells induced by LPS in D-GalN-sensitized mice. The role of HMGB1 in the precise mechanism of apoptotic cell death of hepatocytes in this experimental hepatitis remains to be unknown but is assumed to be implicated in the signal pathways regulating apoptosis [66], [67].

To assess the acetylation of HMGB1 in the inflammatory hepatic specimens, we conducted the immunohistochemical analysis using the antibody to acetylated-lysine. This analysis revealed a few nuclear immunoreactive products in the hepatic cells of control mice. The hepatocytes with nuclear immunoreaction for acetylated-lysine were increased in number 1–3 h after the concomitant administration of LPS/GalN, but they were decreased at 6–8 h. At 8 h, aberrant, extracellular acetylated-lysine expression was labeled and mainly restricted to pericentral inflammatory areas. At 10 h a distinct nuclear expression for acetylated-lysine was again increased in the hepatic cells, although some hepatocytes remained to express cytoplasmic acetylated-lysine in the pericentral areas. Administration of GL strongly inhibited the immunoreaction of acetylated-lysine in the LPS/GalN-induced liver injury. Immunohistochemical analysis for acetylated-lysine used in this experiment shows that administration of GL may inhibit acetylation of lysine in HMGB1 in the liver remnants of the LPS/GalN-induced mice. The ability of GL to suppress ALT levels is observed when administered at 30nmin before or at 10 min and 60 min after LPS/GalN, but GL-treatment has little effect on ALT levels when administered 3 h after LPS/GalN injection [15]. Thus, inhibitory effect of GL on LPS/GalN-induced liver injury might be due to binding directly to HMGB1 [30] before its acetylation. Acetylated HMGB1 has been shown to be involved in regulating HMGB1 DNA binding properties along with its subcellular location [68]. *In vitro* experiments have shown that lysine residues of HMGB1 between 27 and 43 represent functional nuclear localizing signals in macrophages [34]. Also, recent mutation analysis for the abilities of HMGB1 protein to bind and bend DNA has reported the role of lysines 2 and 81 as target sites for acetylation in full-length HMGB1 and truncated tail-less protein, respectively [69]. Serum HMGB1 released following liver ischemia/reperfusion (I/R) *in vivo* is acetylated and hepatocytes exposed to oxidative stress *in vitro* also release acetylated HMGB1 [38]. Levels of acetylated HMGB1 increase with a concomitant decrease in total nuclear histone deacetylase (HDAC) activity, suggesting that suppression in HDAC activity contributes to the increase in acetylated HMGB1 release after oxidative stress in hepatocytes. In their study, the isoforms HDAC1 and HDAC4 were demonstrated as critical in regulating acetylated HMGB1 release. Activation of HDAC1 is decreased in the nucleus of hepatocytes undergoing oxidative stress. In addition, HDAC1 knockdown with siRNA promotes HMGB1 translocation and release. Furthermore, HDAC4 is shuttled from the nucleus to cytoplasm in response to oxidative stress, resulting in decreased HDAC activity in the nucleus. On the contrary, in the present experiment we did not find a decrease in both the cytoplasmic and nuclear HDAC activities after injection of LPS/GalN. In addition, administration of GL did not induce a significant increase of nuclear HDAC activity. Phosphorylation of HMGB1 is another regulatory mechanism that influences its subcellular location [33]. Recently, it was shown that HMGB1 phosphorylation by calcium/calmodulin protein kinase IV caused nuclear-to-cytoplasmic shuttling and release in LPS-stimulated macrophages [70].

Damaged cells activate innate immunity and recruit inflammatory cells by collectively releasing danger signals known as the damage-associated molecular patterns (DAMPs). One such molecule is HMGB1, which has been implicated as an early mediator of organ damage in ischemia/reperfusion (I/R) injury and hemorrhagic shock, as well as a late mediator of lethality in endotoxic shock [2], [4]. In most cases the cells actively secreting HMGB1 appear to be immune cells such as macrophages, natural killer cells [71], and dendritic cells [63]. However, it is becoming increasingly clear that non-immune parenchymal cells also participate in active HMGB1 secretion [72]. It has also been shown that neutralizing antibodies to HMGB1 amelliorate liver I/R injury, thereby suggesting a therapeutic benefit of blocking active HMGB1 release to minimize I/R-associated damage. In contrast, our preliminary work presented the results that the intravenous injection of HMGB1 (500 ng/mouse) 6 hours after LPS/GalN-treatment or HMGB1 alone did not precipitate ALT/AST activity (Fig. S2). Furthermore, the intravenous injection of neutralizing antibodies to HMGB1 (2 mg/kg) did not ameliorate an increase in serum ALT/AST activity in LPS/GalN-injured mice (Fig. S3). In addition to these findings, the expression levels of *Tlr4* mRNA in LPS/GalN-induced hepatic injury were significantly increased compared to the control, but those of *Rage* mRNA conversely decreased in this hepatic injury. Considering together with these data, acute hepatic injury stimulated by a single injection of LPS/GalN might not be caused by HMGB1 released into the extracellular milieu from non-hematopoietic cells (hepatocytes) or activated macrophages/dendritic cells.

In the current investigation, numerous apoptotic cells were observed in the pericentral areas of LPS/GalN-treated liver. To understand mechanisms of hepatocyte apoptosis promoted in the LPS/GalN-induced hepatic injury, we explored the fluctuation in the expression of genes associated with the regulation of apoptotic cell death using the gene microarray analysis (Fig. S1). In this study, the clinical syndrome observed in LPS/GalN-treated animals, associated with diffuse hepatocyte apoptosis, was prevented by administration of GL. On the base of the results obtained from microarray analysis, furthermore, the expression patterns of 4 genes, *Pdcd6*, *Gsto1*, *Cyp2d10*, and *Stc1*, influenced by injecting LPS/GaIN or LPS/GaIN with GL were examined by quantitative PCR using the cDNA from control, LPS/GaIN-injected, and GL+LPS/GaIN-treated liver remnants. To examine the binding of HMGB1 protein to these genes, we performed analysis with ChIP assay and revealed that HMGB1 protein intensely bound to *Gsto1* promoter sequence in LPS/GaIN-injected liver injury but could not detect a combination of HMGB1 with the promoter sequence of other genes. Furthermore, an administration of GL strongly inhibited the binding of HMGB1 to *Gsto1* promoter sequence. GSTs (glutachione transferase) are a superfamily of ubiquitous intracellular enzymes that catalyze the conjugation of glutathione to a wide range of exogenous and endogenous compounds, including chemical carcinogens, therapeutic drugs and oxidative stress products. New members of the GST structural family with novel catalytic activities and functions have recently been discovered [73], [74]. Mouse GSTO1 was indeed identified because of its overexpression in a mouse lymphoma cell line presenting with resistance against a variety of chemo-therapeutics [75]. After transfection with *Gsto1* siRNA, transfected HeLa cells present with a marked increase of apoptosis as compared with controls [76], suggesting that GSTO1 plays an anti-apoptotic role. GSTO1 overexpression is associated with activation of survival pathways including Akt kinase (cell survival factor) and extracellular signal-regulated kinase (ERK) 1/2 (cell survival and proliferation factor) and anti-apoptotic pathways of c-Jun N-terminal kinase (JNK) 1 (apoptosis blockade factor). Immunohistochemistry of LPS-treated liver remnants showed the remarkable decrease of nuclear reactivity and sparse reactivity of cytoplasm with the antibody to activated p65 at 8 or 10 h compared with controls or GL-treated remnants (Fig. S4). Although the activation of NF-κB is associated with potent inflammatory responses in hepatic injury, key roles for this factor in inhibition of apoptosis in the liver have been also demonstrated definitively experimentally [77], [78]. In conclusion, in mouse liver injury induced by a single injection of LPS/GalN, HMGB1 protein appears to be implicated in the (direct and/or indirect) inhibition of a signal pathway associated with anti-apoptosis, i.e. down-regulation of GSTO1, and to stimulate apoptosis of hepatocytes rather than to act as pro-inflammatory factors in this experimental model. However, we cannot exclude the possibility that additional LPS-treatment induces release of HMGB1 into extracellular milieu and precipitates hepatic inflammation through the receptors of TLR4 and/or RAGE. In addition to the role of GL disclosed in this study, it has been reported that GL treatment inhibits the proliferation and migration of cells stimulated by HMGB1 cytokine, as well as HMGB1-induced formation of blood vessels and reduces inflammatory condition [31].

## Supporting Information

Figure S1 Gene microarray analysis. A microarray analysis shows that the expression patterns of 4 genes, *Pdcd6, Gsto1*, *Cyp2d10*, and *Stc1*, are influenced by injecting LPS/GaIN or LPS/GalN with GL.
(TIF)

Figure S2 Serum ALT/AST levels after an injection of HMGB1. An intravenous injection of HMGB1 (500 ng/mouse) alone does not stimulate ALT/AST activity compared with the control. The serum ALT/AST activity is hardly affected compared to LPS/GalN-treatment alone, when HMGB1 (500 ng/mouse) is intravenously injected 6 h after LPS/GalN-treatment.
(TIF)

Figure S3 Effect of antibodies to HMGB1 on mice treated with LPS/GalN. When neutralizing antibodies (2 mg/kg) to HMGB1 are intravenously injected 5.5 h after LPS/GalN-treatment, they do not ameliorate an increase in serum ALT/AST activity induced in LPS/GalN-treated liver injury.
(TIF)

Figure S4 Expression pattern of NF-κB in LPS/GalN-treated mice. Immunohistochemical assessment of LPS/GalN-treatment on the activation of NF-κB in the liver remnants at 0 (**a**), 3 (**b**), 6 (**c**), 8 (**d**), and 10 hours (**e**) after injury and of the inhibitory effects by an administration of GL. At 3 h after LPS-treatment, the stronger nuclear immunoreaction is labeled compared with controls using an antibody against activated p65 (**b**), but its nuclear immunoreaction is gradually reduced in time-dependent manner (**c**, **d**). Immunohistochemistry for activated p65 shows an increase of nuclear reactivity in GL-treated remnants (**f**). *CV*: central vein. *Bars*  = 200 μm.
(TIF)
