# Supplementary figures and images for: Apoptotic Response through a High Mobility Box 1 Protein-Dependent Mechanism in LPS/GalN-Induced Mouse Liver Failure and Glycyrrhizin-Mediated Inhibition
Source: PLoS One. 2014 Apr 1;9(4):e92884. doi: 10.1371/journal.pone.0092884 (PMC3972228; doi:10.1371/journal.pone.0092884)

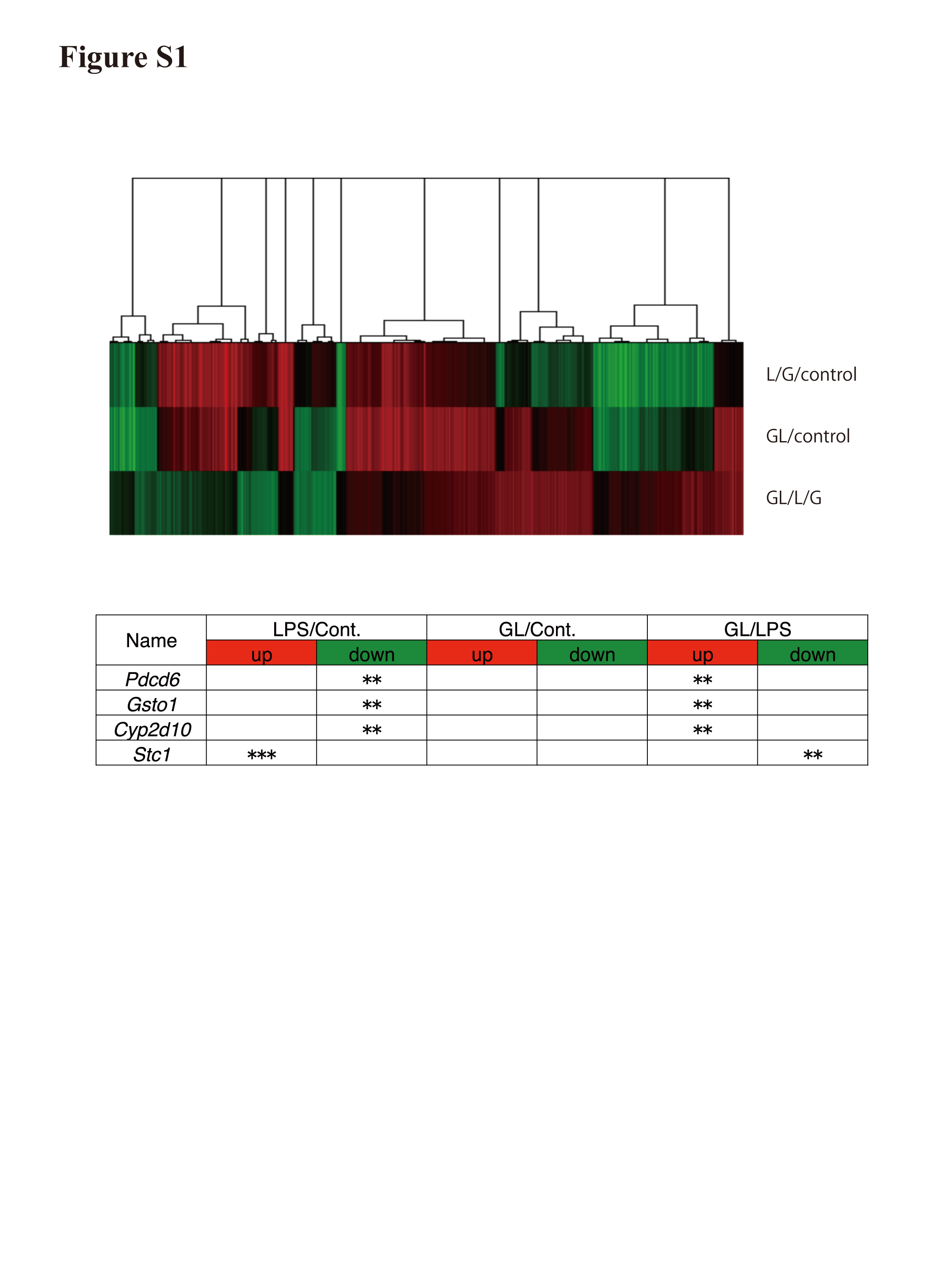

Supplement: Figure S1 — Gene microarray analysis. A microarray analysis shows that the expression patterns of 4 genes, Pdcd6, Gsto1, Cyp2d10, and Stc1, are influenced by injecting LPS/GaIN or LPS/GalN with GL. (TIF) [file pone.0092884.s001.tif]

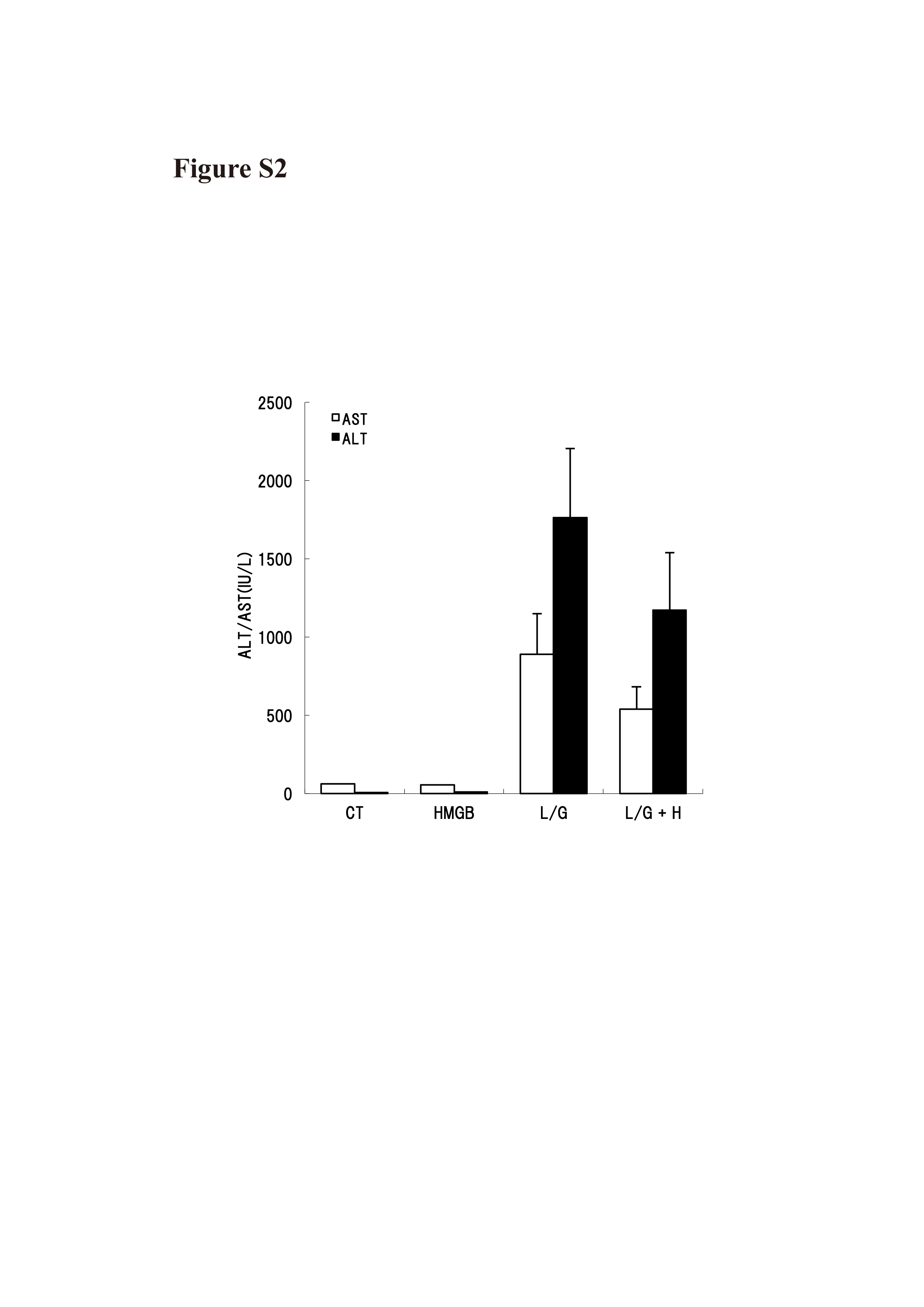

Supplement: Figure S2 — Serum ALT/AST levels after an injection of HMGB1. An intravenous injection of HMGB1 (500 ng/mouse) alone does not stimulate ALT/AST activity compared with the control. The serum ALT/AST activity is hardly affected compared to LPS/GalN-treatment alone, when HMGB1 (500 ng/mouse) is intravenously injected 6 h after LPS/GalN-treatment. (TIF) [file pone.0092884.s002.tif]

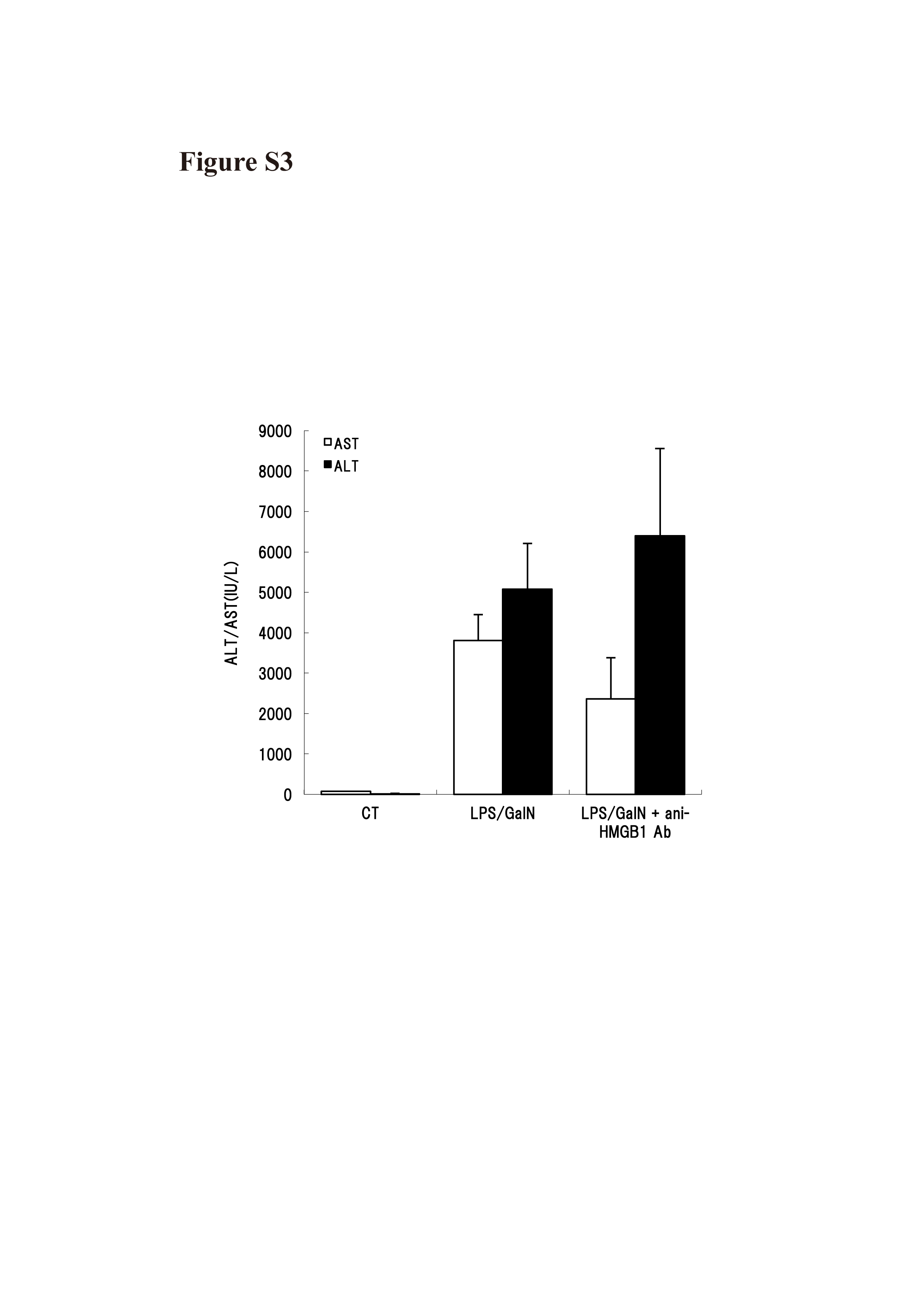

Supplement: Figure S3 — Effect of antibodies to HMGB1 on mice treated with LPS/GalN. When neutralizing antibodies (2 mg/kg) to HMGB1 are intravenously injected 5.5 h after LPS/GalN-treatment, they do not ameliorate an increase in serum ALT/AST activity induced in LPS/GalN-treated liver injury. (TIF) [file pone.0092884.s003.tif]

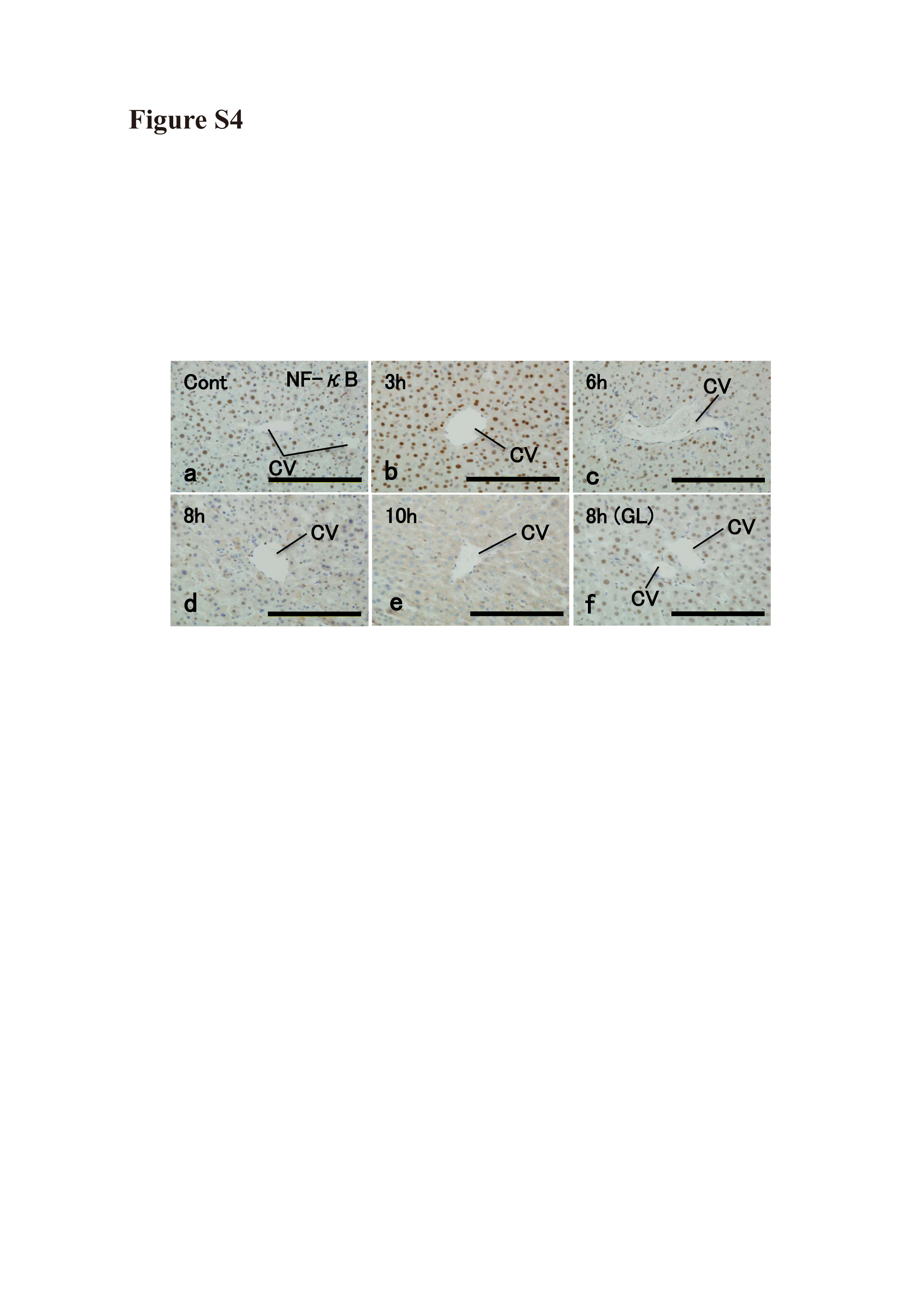

Supplement: Figure S4 — Expression pattern of NF-κB in LPS/GalN-treated mice. Immunohistochemical assessment of LPS/GalN-treatment on the activation of NF-κB in the liver remnants at 0 (a), 3 (b), 6 (c), 8 (d), and 10 hours (e) after injury and of the inhibitory effects by an administration of GL. At 3 h after LPS-treatment, the stronger nuclear immunoreaction is labeled compared with controls using an antibody against activated p65 (b), but its nuclear immunoreaction is gradually reduced in time-dependent manner (c, d). Immunohistochemistry for activated p65 shows an increase of nuclear reactivity in GL-treated remnants (f). CV: central vein. Bars = 200 μm. (TIF) [file pone.0092884.s004.tif]
